# Nonasymptotic Upper Bounds on Binary Single Deletion Codes via Mixed Integer Linear Programming

**DOI:** 10.3390/e21121202

**Published:** 2019-12-06

**Authors:** Albert No

**Affiliations:** Department of Electronic and Electrical Engineering, Hongik University, Seoul 04066, Korea; albertno@hongik.ac.kr; Tel.: +82-2-320-1649

**Keywords:** deletion channel, maximum independent set, mixed integer programming, Varshamov–Tenengolts code

## Abstract

The size of the largest binary single deletion code has been unknown for more than 50 years. It is known that Varshamov–Tenengolts (VT) code is an optimum single deletion code for block length n≤10; however, only a few upper bounds of the size of single deletion code are proposed for larger *n*. We provide improved upper bounds using Mixed Integer Linear Programming (MILP) relaxation technique. Especially, we show the size of single deletion code is smaller than or equal to 173 when the block length *n* is 11. In the second half of the paper, we propose a conjecture that is equivalent to the long-lasting conjecture that “VT code is optimum for all *n*”. This equivalent formulation of the conjecture contains small sub-problems that can be numerically verified. We provide numerical results that support the conjecture.

## 1. Introduction

A deletion channel is one of the most important channels in the history of communication. The channel has a deletion error where the symbol is being removed without knowing the position of it. Unlike many other channels where the positions of symbols remain the same, the decoder needs to specify the position of each symbol, which is called a synchronization issue. Mainly due to this issue, the deletion channel is surprisingly hard to analyze.

There are several different mathematical problem formulations of the deletion channel. One natural way is the probabilistic approach where the deletion occurs in i.i.d. manner with probability *p*. Kanoria and Montanari provided an approximation of the channel capacity of binary deletion channel when p→0 [1]. However, the channel capacity is unknown in this setting even in binary case.

Alternatively, we can define the problem in algebraic way. We assume that there will be at most *k* deletion errors while transmitting *n* number of symbols. The question is the maximum size of the deletion code that can correct any *k* deletion errors. There is a nice survey paper by Sloane [2], and it is easy to see that the problem is extremely challenging even in single deletion case.

Although the problem is still open, there has been some progress in this algebraic setting. In binary case, Varshamov and Tenengolts proposed a simple code (VT-code) construction that corrects any single deletion error [3]. It is asymptotically optimum when *n* grows while the number of deletions k=1 is fixed. VT-code is known to be optimum when n≤10 and conjectured that it is optimum for all *n*. Tenengolts generalized VT-code to a non-binary version [4]. Gabrys and Sala proposed a code that can correct two deletions [5]. Sima and Bruck also generalized VT-code that can correct *k* deletions [6]. Both results [5,6] show n−log|C|=O(klogn) where C is the deletion code. This implies that they achieved order optimal redundancy where the optimality is shown by Levenshtein [7]. However, their works do not specify the coefficient of klogn term while VT code satisfies n−log|C|=logn+o(logn) in the single deletion case.

VT-code naturally suggests a lower bound of the size of the optimum single deletion code in binary case, but the upper bounds are rarely provided. Levenshtein found an analytic formula for upper bounds [8]. Kulkarni and Kiyavashi proposed better upper bounds for any number of deletions and any size of alphabet [9]. Cullina and Kiyavashi refined Levenshtein’s upper bound [10].

There are attempts to find nonasymptotic upper bounds numerically for small *n*. The most popular approach is using graph theory, which is based on the fact that the optimum single deletion code problem has an equivalent formulation of maximum independent set problem. In this formulation, the size of the largest single deletion code is equal to the size of the maximum independent set. A well-known upper bound of the maximum independent set is Lovász theta number, which can be obtained by solving Semidefinite Programming (SDP) relaxed problem [11]. Upper bounds from Lovász theta number are tight for n≤8; in other words, VT-code is optimum when the block length *n* is smaller than 8. Butenko et al. provided a numerical approach to bound the size of maximum independent set [12], and showed that VT-code is optimum when the block length is n=9. Kulkarni et al. proposed a bounding technique using Linear Programming (LP) relaxation of the graph problem [13]. This bound is weaker than that of Lovász’s, but the complexity is lower so that we can compute the bounds for larger block lengths *n*. For recent progress, we refer to Sloane’s webpage [14], which also mentions that VT-code is optimum when the block length is n=10.

In this paper, we propose a new method that provides an improved nonasymptotic upper bound. We partially relax the graph problem, and obtain Mixed Integer Linear Program (MILP) where some of variables are relaxed but some of them are not. MILP has reasonably low complexity and provides a better bound. For example, when the block length is n=11, we show the size of optimum single deletion code is less than or equal to 173, where the SDP provides a bound of 174.

We also find an equivalent formulation of the conjecture that “VT-code is optimum”. This equivalent form consists of several small sub-conjectures where VT-code is optimum if and only if all those sub-conjectures are true. The advantage of this equivalent conjecture is that we can numerically verify the sub-conjectures using (Mixed) Integer Programming. Note that we can disprove the original conjecture if any of sub-conjectures are not true. We numerically verified some of sub-conjectures for n≤16. This does not prove or disprove the optimality of VT-code, but it supports the original conjecture.

The remainder of the paper is organized as follows. In Section 2, we revisit some of the known results of single deletion codes. Section 3 presents the new bounding technique using Mixed Integer Linear Programming with improved upper bound. In Section 4, we present an equivalent formula of the conjecture as well as some numerical supports. Finally, we conclude in Section 5.

### Notation

Let X={0,1} be a set of binary alphabet. We denote a *n*-tuple using super script, i.e., $x^{n}=x_{1}x_{2}\cdots x_{n}\in X^{n}$. In addition, let $0^{n}$ be an all zero vector. For clarity, we use $x^{n}\in X^{n}$ for an *n*-dimensional binary vector, and $y^{n+1}\in X^{n+1}$ for an n+1-dimensional binary vector. We also use concatenation of binary vectors. For example, $x^{n}0$ is an n+1-dimensional binary vector where the last bit is 0.

## 2. Preliminaries

### 2.1. Single Deletion Code

Define a deletion ball $B_{D}\left(x^{n}\right)\subset X^{n-1}$, which is a collection of (n−1)-dimensional binary vectors that are one-bit deleted version of $x^{n}$. We can define an insertion ball $B_{I}\left(x^{n}\right)\subset X^{n+1}$ in a similar manner.

**Definition** **1.** For a positive integer n, a set of binary vectors $C\subset X^{n}$ is a single deletion code if $B_{D}\left(c_{1}\right)\cap B_{D}\left(c_{2}\right)=\emptyset$ for all $c_{1}\neq c_{2}$ in C.

The definition implies that the single deletion code can always correct a single deletion error. The following lemma shows that the single deletion code can also correct a single insertion error.

**Lemma** **1.** ([13], Lemma 2.1) A set of binary vectors $C\subset X^{n}$ is a single deletion code if and only if $B_{I}\left(c_{1}\right)\cap B_{I}\left(c_{2}\right)=\emptyset$ for all $c_{1}\neq c_{2}$ in C.

The above lemma is simply from the fact that $B_{I}\left(c_{1}\right)\cap B_{I}\left(c_{2}\right)\neq \emptyset$ if and only if $B_{D}\left(c_{1}\right)\cap B_{D}\left(c_{2}\right)\neq \emptyset$.

### 2.2. Varshamov–Tenengolts Codes

Let $v_{n}:X^{n}\to \mathbb{Z}$ be a function that computes the “VT-weights” of an *n*-dimensional binary vector.

$$\begin{array}{c} v_{n}\left(x^{n}\right)=\sum_{k=1}^{n}k\cdot x_{k}. \end{array}$$

Note that we do not take any modulo operations, and therefore $v_{n}\left(x^{n}\right)$ can take value from 0 to $\frac{n(n+1)}{2}$.

For 0≤a≤n, VT-code [3] is defined by

$$\begin{array}{c} VT_{a}\left(n\right)=\left\{x^{n}\in X^{n}:v_{n}\left(x^{n}\right)\equiv a\;\left(\mathrm{mod}\;n+1\right)\right\} \end{array}$$

which is a single deletion code [7,15]. Levenshtein showed that $VT_{a}\left(n\right)$ is perfect for all 0≤a≤n [16]. In other words,

$$\begin{array}{c} X^{n-1}=\underset{x^{n}\in VT_{a}\left(n\right)}{⋃}B_{D}\left(x^{n}\right). \end{array}$$

For any *a*, we have $|VT_{0}\left(n\right)|\geq |VT_{a}\left(n\right)|\geq |VT_{1}\left(n\right)|$ where the first inequality is from Varshamov [3] and the second inequality is from Ginzburg [17]. Thus, the size of the optimum single deletion code is lower bounded by $|VT_{0}\left(n\right)|$. An analytic formula $|VT_{0}\left(n\right)|$ is given in [2]:

$$\begin{array}{c} |VT_{0}\left(n\right)|=\frac{1}{2(n+1)}\sum_{\mathrm{odd}\;d|n+1}\phi \left(d\right)2^{(n+1)/d} \end{array}$$

where ϕ(d) is Euler’s totient function.

Borchers showed that $VT_{0}\left(n\right)$ is optimum single deletion code for n≤10 [14]. The optimality of VT-code is still open for n≥11. In the case of n=11, the size of VT-code is $|VT_{0}\left(n\right)|=172$ but the best known upper bound of the largest single deletion code is |C|≤174.

### 2.3. Maximum Independent Set Approach

Consider a graph where all binary vectors are nodes. There exists an edge between two nodes $x^{n}$ and ${\tilde{x}}^{n}$ if and only if $B_{D}\left(x^{n}\right)\cap B_{D}\left({\tilde{x}}^{n}\right)\neq \emptyset$. Then, the optimum single deletion code corresponds to the maximum independent set. Note that the Maximum Independent Set (MIS) problem is NP-complete.

There are reduction rules in graph theory that provide an equivalent graph problem while reducing the size of the graph. Isolated vertex removal technique is useful in our case. An isolated vertex is a node for which its neighborhood forms a clique. For example, in our case, the neighborhood set of node $0^{n}$ is {100⋯00,010⋯00,…,00⋯01}, and {00⋯00,100⋯00,010⋯00,…,00⋯01} forms a clique. This implies that the node $0^{n}$ is an isolated vertex. Butenko et al. showed that there exists a maximum independent set that contains all isolated vertices [12]. Thus, there exists an optimum single deletion code that contains both $0^{n}$ and $1^{n}$.

Note that it is still infeasible to solve our MIS problem with state of the art algorithms [18,19] when n≥11. Segundo et al. proposed a variation of BBMC [20] and found the maximum independent set of the graph induced from two deletion (k=2) channel [14]. However, the graph induced from single deletion channel (k=1) is more challenging to find the maximum independent set.

### 2.4. LP Relaxation

For simplicity, we define several functions and new notations. Let $N=2^{n}$, and [N−1]={0,1,…,N−1} be the set that contains all nonnegative integers smaller than or equal to N−1. We further let $b_{n}:\left[N-1\right]\to {\left\{0,1\right\}}^{n}$ be the function that converts the decimal number to the binary vector (e.g., $b_{4}\left(3\right)=0011$). Note that we drop *n* if it is clear from the context, i.e., $b\equiv b_{n}$.

In the above section, we define a graph where there exists an edge between $x^{n}$ and ${\tilde{x}}^{n}$ if their deletion balls share an element. Instead, we define an equivalent graph where the set of nodes are V=[N−1]. The set of edges E⊂V×V is derived naturally where (i,j)∈E if and only if there is an edge between b(i) and b(j).

Our goal is to find an independent set U⊂V that has maximum number of elements. For 0≤i≤N−1, let $X=(X_{0},X_{1},\ldots ,X_{N-1})$ be binary variables where $X_{i}=1$ if i∈U and $X_{i}=0$ if i∉U. If there exists an edge between *i* and *j*, then the independent set U cannot contain both *i* and *j*. Thus, the following Integer Programming (IP) problem is equivalent to the maximum independent set problem.

$$\begin{array}{cc} \max_{X}\; & \sum_{i=0}^{N-1}X_{i}\\ s.t.\; & X_{i}+X_{j}\leq 1,\;\mathrm{for}\;(i,j)\in E\\ \; & X_{i}\in \left\{0,1\right\},\;\;i=0,1,\ldots ,N-1. \end{array}$$

Clearly, it is an NP-hard problem, which is extremely challenging to solve. Instead, we can relax it to an easier problem, and bounding the solution of the IP. One way of doing it is classical Linear Programming (LP) relaxation, which is given by

$$\begin{array}{cc} \max_{X}\; & \sum_{i=0}^{N-1}X_{i}\\ s.t.\; & X_{i}+X_{j}\leq 1,\;\mathrm{for}\;(i,j)\in E\\ \; & 0\leq X_{i}\leq 1,\;\;i=0,1,\ldots ,N-1. \end{array}$$

LP relaxation allows the variable $X_{i}$ to take a value between 0 and 1, and the solution of relaxed problem provides an upper bound of the original IP. However, this gives a trivial solution in our case, which is $X_{i}=1/2$ for all i∈{0,1,…N−1}. The maximum value of the objective function is $2^{n-1}$, and it is much larger than the known upper bound $\frac{2^{n}-2}{n-1}$ [9].

Kulkarni et al. proposed another LP relaxation [9]. The idea is that the independent set can contain at most one node from each clique. For any (n−1)-dimensional vector $y^{n-1}$, the set of nodes $Q_{I}\left(y^{n-1}\right)\overset{\Delta }{=}\left\{i:b\left(i\right)\in B_{I}\left(y^{n-1}\right)\right\}$ forms a clique since $y^{n-1}\in B_{D}\left(b\left(i\right)\right)\cup B_{D}\left(b\left(j\right)\right)$ for all $i,j\in Q_{I}\left(y^{n-1}\right)$. Thus, the independent set U can take at most one node from $Q_{I}\left(y^{n-1}\right)$, and we have *clique constraints* $\sum_{i\in Q_{I}\left(y^{n-1}\right)}X_{i}\leq 1$. This implies another LP relaxation which provides a tighter upper bound of the original IP problem.

$$\begin{array}{cc} \max_{X}\; & \sum_{i=0}^{N-1}X_{i}\\ s.t.\; & \sum_{i\in Q_{I}\left(y^{n-1}\right)}X_{i}\leq 1,\;\;\mathrm{for}\;y^{n-1}\in X^{n-1}\\ \; & 0\leq X_{i}\leq 1,\;\;\;i=0,1,\ldots ,N-1. \end{array}$$

On the other hand, Lovász proposed an SDP-relaxation of the Maximum Independent Set (MIS) problem [11]. The solution of SDP problem is called Lovász theta number, which provides a tighter bound of MIS problem.

The following table presents the upper bound from the above relaxations as well as $|VT_{0}\left(n\right)|$ which is a lower bound.
n**6****7****8****9****10****11****12**$|VT_{0}\left(n\right)|$1016305294172316Lovász10.0016.8430.0053.0395.98174.73-LP10.2517.1530.3253.5696.52175.19321.27

Note that the complexity of LP relaxed problem is low, and we can get bounds for n≥13 as well. For example, the size of maximum deletion code is smaller than or equal to 593 when n=13. However, due to the complexity issue, we are not able to compute Lovász theta number for n≥12. For example, it took more than 24 h on our machine when n=12.

## 3. Mixed Integer Linear Programming

LP is faster than the original IP problem; however it is hard to parallelize. On the other hand, IP inherently uses branch-and-bound technique which can be parallelized, but still intractable with current multi-thread processors. In this section, we propose a Mixed Integer Linear Programming (MILP) problem, which is in between LP problem and the original IP problem.

### 3.1. Main Results

In the LP relaxation, all variables are relaxed as described in Section 2.4. Instead, we relax specific variables only, which provides a semi-relaxed optimization problem. More precisely, we design S⊂V and keep $X_{i}$ to be binary variable for i∈S while other variables are relaxed as in LP relaxation. This provides a Mixed Integer Programming (MIP) problem where variables are either integer or real numbers:

$$\begin{array}{cc} \max_{X}\; & \sum_{i=0}^{N-1}X_{i}\\ s.t.\; & \sum_{i\in Q_{I}\left(y^{n-1}\right)}X_{i}\leq 1,\;\;\mathrm{for}\;y^{n-1}\in X^{n-1}\\ \; & X_{i}\in \left\{0,1\right\},\;\;\;\mathrm{for}\;i\in S\\ \; & 0\leq X_{i}\leq 1,\;\;\;\mathrm{for}\;i\notin S\\ \; & X_{0}=1\\ \; & X_{N-1}=1. \end{array}$$

The last two constraints are because there exists a maximum independent set that contains both $0^{n}$ and $1^{n}$ from Section 2.3.

MIP is generally as computationally demanding as IP problems. However, since all constraints are linear, the above optimization problem is a Mixed Integer Linear Programming (MILP) problem. MILP can be solved in reasonable amount of time if we carefully design the set S. Let $MILP_{n}\left(S\right)$ denote the above MILP problem.

If S=∅, then $MILP_{n}\left(S\right)$ is equivalent to LP. On the other extreme, if S=V, then $MILP_{n}\left(S\right)$ is equivalent to the original IP. If S is nontrivial subset of $X^{n}$, then the solution of $MILP_{n}\left(S\right)$ provides a tighter upper bound of maximum independent set problem while having low complexity.

Clearly, we prefer smaller S because of complexity. Thus, the goal is designing S in smart way. The main idea is increasing the size of S in greedy manner. We start from fully relaxed LP problem, i.e., S=∅, and add elements one by one under certain criterion.

More precisely, we solve $MILP_{n}\left(S\right)$ in each iteration and add a node *i* to S based on the following rule. Let $d:\left[N-1\right]\to \left\{0,1,\ldots ,2^{n-1}\right\}$ be the function which indicates the number of *clique constraints* that contains *i*. Since $i\in Q_{I}\left(y^{n-1}\right)$ if and only if $y^{n-1}\in B_{D}\left(b\left(i\right)\right)$, we have $d\left(i\right)=|B_{D}\left(b\left(i\right)\right)|$. Thus, the variable $X_{i}$ affects the d(i) number of clique constraints. Furthermore, if $X_{i}$ is large, it restricts other variables in *clique constraints* more. Thus, we measure the amount of “impact” of variable $X_{i}$ by $d\left(i\right)\times X_{i}$. Finally, the algorithm finds the node *i* that maximizes $d\left(i\right)\times X_{i}$ and add it to S. This procedure is described in Algorithm 1.
**Algorithm 1** Sequential MILP. **Input: target threshold**
τ **Output: new bound**
*T***procedure** S_EQ_MILP(τ)  Set S=∅, and T=inf  **do**   Solve $MILP_{n}\left(S\right)$ and let *T* be the objective function value and *X* be the solution   $i_{0}=\arg\max_{i\notin S}d\left(i\right)\times X_{i}$   $S\leftarrow S\cup \{i_{0}\}$  **while**
|S|<N and T≥τ  **return**
*T***end procedure**

The above algorithm takes a target threshold τ as an input which can be a previously known upper bound of the original IP. In each iteration, it computes the objective function value *T* of $MILP_{n}\left(S\right)$. Whenever *T* is smaller than the target bound τ, then the algorithm halts and we get a new bound *T* of the size of maximum independent set. For example, suppose we let $\tau =|VT_{0}\left(n\right)|+1$ and the above program halts with T<τ, then we have a new upper bound that the size of the maximum single deletion code is strictly smaller than $|VT_{0}\left(n\right)|+1$. In such case, we can claim that $VT_{0}\left(n\right)$ is the optimum single deletion code. On the other hand, suppose the program ends with |S|=N which means S=V. In such case, the return value *T* is the size of the maximum independent set because it is the objective function value of the original IP. Note that the size of S is increased by 1 in each iteration, and therefore there will be at most *N* iterations.

Note that the way of choosing $i_{0}=\arg\max_{i\notin S}d\left(i\right)\times X_{i}$ is not an optimum way. However, it is an effective way, as shown in Section 3.2.

### 3.2. Experiments

We implemented Python code using PULP python package [21] with cbc solver [22]. Note that cbc solver supports multiple threads. For our experiments, we used a machine with AMD Threadripper 1950X processor and 64 GB of RAM. The operating system was Ubuntu 18.04 LTS.

In the case of n=11, for any single deletion code C, we have an upper bound |C|≤174 from Lovász theta number. LP provides a weaker upper bound |C|≤175. However, the proposed MILP problem $MILP_{n}\left(S\right)$ provides |C|≤174.993 when

$$\begin{array}{cc} S=\{ & 216,1702,1236,1140,1678,1050,154,105,538,\\ \; & 1701,1941,532,907\}. \end{array}$$

Note that we convert the binary vectors to decimal numbers for simplicity (e.g., 00011011000→216). This implies |C|≤174 which is the same upper bound from Lovász number, but obtained more efficiently.

More interestingly, $MILP_{n}\left(S\right)$ provides |C|≤173.988 when

$$\begin{array}{cc} S=\{ & 216,1702,1236,1140,1678,1050,154,105,538,\\ \; & 1701,1941,532,907,1335,1194,1515,1942,1366,\\ \; & 688,1200,1365,682,854,778,340,576,847,\\ \; & 1851,853,1300,537,322,70,681,556,1359,\\ \; & 1749,196,1755,1707,1709,1533,1371,1539,667,\\ \; & 1306,1750,514,1708,1323,811,724,1706,756,\\ \; & 603,1440,1878,1370,938,1364,683,842,378,\\ \; & 1354,1509,1450,1258,850,598,1373,1494,1482,\\ \; & 1362,553,554,1492,333,1493,674,1498,1386,\\ \; & 874,596,1738,1690,426,1378\}. \end{array}$$

Note that it took 27,096 s to solve the last (88-th) $MILP_{n}\left(S\right)$ with 28 threads. This provides an improved upper bound |C|≤173. Thus, we have the following Corollary.

**Corollary** **1.** For n=11, the size of single deletion code is smaller than or equal to 173.

In case of n=12, for any single deletion code C, an upper bound from LP implies |C|≤321. However, the proposed method $MILP_{n}\left(S\right)$ provides |C|<320.998 when

$$\begin{array}{cc} S=\{ & 3627,1215,360,45,423,4050,2880,3024,1071,\\ \; & 1047,3048,1625,1958,3714,3906\}. \end{array}$$

Thus, we have an improved upper bound |C|≤320.

### 3.3. Connection to Metaheuristics

Although MILP problem has lower complexity than the original IP problem, MILP often encounter the computational issue as well. This is because the most state-of-the-art MILP solvers such as CPLEX [23] are based on branch-and-bound techniques, and it often has exponentially large search space. Thus, the smartly fixing the variable to binary is necessary as we presented in the previous section.

Similar heuristic algorithms appear in various other computationally challenging (Mixed) Integer Programming problems, such as lot sizing problem [24,25] and connected facility location problem [26]. This is commonly referred to as hybrid metaheuristics. For example, Wilbaut and Hanafi proposed iterative idea to solve MIP problems [27]. The authors applied this idea to IP problems such as knapsack [28]. The idea is iteratively solving the LP relaxed problem to get an upper bound and reduced problem with fixing variables to get a lower bound until the lower and upper bounds match. In this paper, we do not fix the value of variable, but remove the relaxed constraints (so that some variables remain binary). For other works in metaheuristics, we refer the interested reader to the nice survey paper by Blum et al. [29].

## 4. Equivalent Conjecture

The above semi-relaxation provides an improved upper bound; however, the running time is still an issue. In this section, we provide smaller optimization problems that can provide insights for the optimality of $VT_{0}\left(n\right)$ code.

### 4.1. VT-Sum Based Partition

For $0\leq i\leq \frac{n(n+1)}{2}$, let $S_{n,i}\subset X^{n}$ be the set of binary vectors whose VT-weights are *i*.

$$\begin{array}{c} S_{n,i}=\left\{x^{n}\in X^{n}:v_{n}\left(x^{n}\right)=i\right\}. \end{array}$$

Clearly, $\left\{S_{n,i}:0\leq i\leq \frac{n(n+1)}{2}\right\}$ is a partition of $X^{n}$, and $\left\{S_{n,i}:0\leq i\leq \frac{n(n+1)}{2},\;i\equiv a\;\left(\mathrm{mod}\;n+1\right)\right\}$ is a partition of $VT_{a}\left(n\right)$.

The following lemma provides useful properties of $S_{n,i}$.

**Lemma** **2.** *Suppose n and $0\leq i,j\leq \frac{n(n+1)}{2}$ are positive integers. Then,*

*1*. *$\left|S_{n,i}|=|S_{n,\frac{n(n+1)}{2}-i}\right|$.*
*2*. *If |i−j|≥n+1, then $B_{D}\left(S_{n,i}\right)\cap B_{D}\left(S_{n,j}\right)=\emptyset$.*
*3*. *If |i−j|≥n+1, then $B_{I}\left(S_{n,i}\right)\cap B_{I}\left(S_{n,j}\right)=\emptyset$.*

**Proof.** 

1. There exists a one-to-one correspondence between $S_{n,i}$ and $S_{n,\frac{n(n+1)}{2}-i}$ which is an element-wise binary complement.
2. For any element $z^{n-1}\in B_{D}\left(S_{n,i}\right)$, it is easy to show that $i-n\leq v_{n-1}\left(z^{n-1}\right)\leq i$. If |i−j|≥n+1, then {i,i−1,…,i−n}∩{j,j−1,…,j−n}=∅, and therefore $B_{D}\left(S_{n,i}\right)\cap B_{D}\left(S_{n,j}\right)=\emptyset$.
3. For any $x^{n},{\tilde{x}}^{n}\in X^{n}$, it is clear that $B_{D}\left(x^{n}\right)\cap B_{D}\left({\tilde{x}}^{n}\right)=\emptyset$ if and only if $B_{I}\left(x^{n}\right)\cap B_{I}\left({\tilde{x}}^{n}\right)=\emptyset$. Thus, the third property is a direct consequence of the second property.

□

**Remark** **1.** *If we view the original problem as a maximum independent set problem, the above partition $S_{n,0},\ldots ,S_{n,\frac{n(n+1)}{2}}$ can be useful since:*

- *There are no internal edges in $S_{n,i}$ for all i.*
- *There are no edges between $S_{n,i}$ and $S_{n,j}$ if |i−j|≥n+1.*

*This is not exactly a “partite” graph but has a similar flavor of it, and there might be an efficient way of finding a maximum independent set.*

### 4.2. Equivalent Conjecture

For a given single deletion code C, we also define a similar partition of C. For $0\leq i\leq \frac{n(n+1)}{2}$, we let $C_{i}=C\cap S_{n,i}=\left\{x^{n}\in C:v_{n}\left(x^{n}\right)=i\right\}$. Then, we are ready to state our first lemma which is a building block of the main conjecture.

**Lemma** **3.** *Let n be a positive integer, and $C\subset X^{n}$ be a single deletion code. If there exists an integer $0\leq k\leq \lfloor \frac{n}{2}\rfloor$ such that*

(1) $$\begin{array}{c} |C_{0}\left|+\right|C_{1}\left|+\ldots +\right|C_{k(n+1)}\left|>\right|S_{n,0}\left|+\right|S_{n,n+1}\left|+\ldots +\right|S_{n,(n+1)k}|, \end{array}$$

*then $VT_{0}\left(n\right)$ is not an optimum single deletion code.*

**Proof.**  Suppose there exists *k* such that $|C_{0}\left|+\right|C_{1}\left|+\cdots +\right|C_{(n+1)k}\left|>\right|S_{n,0}\left|+\right|S_{n,n+1}\left|+\cdots +\right|S_{n,(n+1)k}|$. Then, we can define a new single deletion code

$$\begin{array}{c} \tilde{C}=\left(\overset{(n+1)k}{\underset{i=0}{⋃}}C_{i}\right)\cup \left(\overset{\left\lfloor n/2\right\rfloor }{\underset{j=k+1}{⋃}}S_{n,(n+1)j}\right). \end{array}$$

Clearly, $\tilde{C}$ is a single deletion code since $C_{i}\cap S_{n,(n+1)j}=\emptyset$ for all i≤(n+1)k and j≥k+1. Then, the size of the new single deletion code is given by

$$\begin{array}{cc} |\tilde{C}|= & \sum_{i=0}^{(n+1)k}|C_{i}|+\sum_{j=k+1}^{\left\lfloor n/2\right\rfloor }\left|S_{n,(n+1)j}\right|\\ > & \sum_{j=0}^{k}|S_{n,(n+1)j}|+\sum_{j=k+1}^{\left\lfloor n/2\right\rfloor }\left|S_{n,(n+1)j}\right|\\ = & \sum_{j=0}^{\left\lfloor n/2\right\rfloor }\left|S_{n,(n+1)j}\right|\\ = & |VT_{0}\left(n\right)|. \end{array}$$

This concludes the proof. □

By the first property of Lemma 2, we have $|VT_{0}\left(n\right)|=|VT_{\frac{n+1}{2}}\left(n\right)|$ for odd *n*. Thus, we have the following lemma as well, which is essentially the same as Lemma 3.

**Lemma** **4.** *Let n be an odd positive integer, and $C\subset X^{n}$ be a single deletion code. If there exists an integer $0\leq k\leq \frac{n-1}{2}$ such that*

(2) $$\begin{array}{c} |C_{0}\left|+\right|C_{1}\left|+\ldots +\right|C_{k\left(n+1\right)+\frac{n+1}{2}}\left|>\right|S_{n,\frac{n+1}{2}}\left|+\right|S_{n,n+1+\frac{n+1}{2}}\left|+\ldots +\right|S_{n,\left(n+1\right)k+\frac{n+1}{2}}|, \end{array}$$

*then $VT_{0}\left(n\right)$ is not an optimum single deletion code.*

**Proof.**  Suppose *n* is odd and there exists *k* such that $|C_{0}\left|+\right|C_{1}\left|+\cdots +\right|C_{\left(n+1\right)k+\frac{n+1}{2}}\left|>\right|S_{n,\frac{n+1}{2}}\left|+\right|S_{n,n+1+\frac{n+1}{2}}\left|+\cdots +\right|S_{n,\left(n+1\right)k+\frac{n+1}{2}}|$. Then, we can define a new single deletion code

$$\begin{array}{c} \tilde{C}=\left(\overset{\left(n+1\right)k+\frac{n+1}{2}}{\underset{i=0}{⋃}}C_{i}\right)\cup \left(\overset{\frac{n-1}{2}}{\underset{j=k+1}{⋃}}S_{n,\left(n+1\right)j+\frac{n+1}{2}}\right). \end{array}$$

Again, the size of the new code is given by

$$\begin{array}{cc} \left|\tilde{C}\right| & =|C_{0}|+|C_{1}|+\cdots +|C_{\left(n+1\right)k+\frac{n+1}{2}}|+\sum_{j=k+1}^{\frac{n-1}{2}}\left|S_{n,\left(n+1\right)j+\frac{n+1}{2}}\right|\\ \; & >|S_{n,\frac{n+1}{2}}|+|S_{n,n+1+\frac{n+1}{2}}|+\cdots +|S_{n,\left(n+1\right)k+\frac{n+1}{2}}|\\ \; & =|S_{n,\frac{\left(n+1)(n-1\right)}{2}}|+|S_{n,\frac{\left(n+1)(n-3\right)}{2}}|+\cdots +|S_{n,0}|\\ \; & =|VT_{0}\left(n\right)|, \end{array}$$

This concludes the proof. □

**Conjecture** **1.**

*1*. *For even n, any single deletion code*$C\subset X^{n}$*satisfies*
  $$\begin{array}{c} |C_{0}\left|+\right|C_{1}\left|+\cdots +\right|C_{(n+1)k}\left|\leq \right|S_{n,0}\left|+\right|S_{n,n+1}\left|+\cdots +\right|S_{n,(n+1)k}| \end{array}$$
  *for all*$0\leq k\leq \frac{n}{2}$.
*2*. *For odd n, any single deletion code*$C\subset X^{n}$*satisfies*
  $$\begin{array}{c} |C_{0}\left|+\right|C_{1}\left|+\cdots +\right|C_{(n+1)k+(n+1)/2}\left|\leq \right|S_{n,(n+1)/2}\left|+\right|S_{n,n+1+(n+1)/2}\left|+\cdots +\right|S_{n,(n+1)k+(n+1)/2}| \end{array}$$
  *for all*$0\leq k\leq \frac{n-1}{2}$.

The following theorem tells us that the above conjecture is equivalent to the original conjecture that “VT-code is optimum”.

**Theorem** **1.** $VT_{0}\left(n\right)$ code is an optimum single deletion code if and only if Conjecture 1 holds.

**Proof.**  Note that the “only if” part (for both even and odd *n*) directly comes from Lemmas 3 and 4.

Suppose *n* is even and $|C_{0}\left|+\right|C_{1}\left|+\cdots +\right|C_{(n+1)k}\left|\leq \right|S_{n,0}\left|+\right|S_{n,n+1}\left|+\cdots +\right|S_{n,(n+1)k}|$ for all *k*. If we let *k* be n/2, then we have

$$\begin{array}{cc} |C|= & |C_{0}\left|+\right|C_{1}\left|+\cdots +\right|C_{(n+1)n/2}|\\ \leq & |S_{n,0}\left|+\right|S_{n,n+1}\left|+\cdots +\right|S_{n,(n+1)n/2}|\\ = & |VT_{0}\left(n\right)|. \end{array}$$

Suppose *n* is odd and $|C_{0}\left|+\right|C_{1}\left|+\cdots +\right|C_{(n+1)k+(n+1)/2}\left|\leq \right|S_{n,(n+1)/2}\left|+\right|S_{n,n+1+(n+1)/2}\left|+\cdots +\right|S_{n,(n+1)k+(n+1)/2}|$ for all *k*. If we let *k* be (n−1)/2, then we have

$$\begin{array}{cc} |C|= & |C_{0}\left|+\right|C_{1}\left|+\cdots +\right|C_{(n+1)n/2}|\\ \leq & |S_{n,(n+1)/2}\left|+\right|S_{n,n+1+(n+1)/2}\left|+\cdots +\right|S_{n,(n+1)n/2}|\\ \leq & |S_{n,(n+1)(n-1)/2}\left|+\right|S_{n,(n+1)(n-3)/2}\left|+\cdots +\right|S_{n,0}|\\ = & |VT_{0}\left(n\right)|. \end{array}$$

□

### 4.3. Special Case of k=1

Theorem 1 implies that if we can find any code that satisfies the inequality in Equation (1) or the inequality in Equation (2), then VT code is not optimum. Thus, the plausible strategy to disprove the conjecture is finding a counterexample for Conjecture 1. However, in this section, we show the inequality in Equation (1) is true for all *n* when k=1.

We define two functions that are useful in the remaining sections. The following lemma is for the definition of the first function.

**Lemma** **5.** *For any n-dimensional binary vector $x^{n}$,*

$$\begin{array}{c} v_{n+1}\left(B_{I}\left(x^{n}\right)\right)\overset{\Delta }{=}\left\{v_{n+1}\left({\tilde{x}}^{n+1}\right):{\tilde{x}}^{n+1}\in B_{I}\left(x^{n}\right)\right\}=\left\{v_{n}\left(x^{n}\right),v_{n}\left(x^{n}\right)+1,\ldots ,v_{n}\left(x^{n}\right)+n+1\right\}. \end{array}$$

**Proof.**  It is well-known that the size of $B_{I}\left(x^{n}\right)$ is n+2 for all $x^{n}$ [30]. Clearly, a single insertion can only increase a VT-sum, in other words, for $y^{n+1}\in B_{I}\left(x^{n}\right)$,

$$\begin{array}{c} v_{n+1}\left(y^{n+1}\right)\geq v_{n}\left(x^{n}\right). \end{array}$$

On the other hand, a single insertion can increase a VT-sum by at most n+1 by adding “1” to the last position. More precisely, for $y^{n+1}\in B_{I}\left(x^{n}\right)$, we have

$$\begin{array}{c} v_{n+1}\left(y^{n+1}\right)\leq v_{n}\left(x^{n}\right)+n+1=v_{n+1}\left(x^{n}1\right). \end{array}$$

Note that $\left\{v_{n+1}\left(y^{n+1}\right):y^{n+1}\in B_{I}\left(x^{n}\right)\right\}$ are all distinct, and therefore

$$\begin{array}{c} v_{n+1}\left(B_{I}\left(x^{n}\right)\right)=\left\{v_{n}\left(x^{n}\right),v_{n}\left(x^{n}\right)+1,\ldots ,v_{n}\left(x^{n}\right)+n+1\right\}. \end{array}$$

□

First, we define a map *g* which maps $x^{n}$ to $y^{n+1}$ where $v_{n+1}\left(y^{n+1}\right)\equiv 0\;\mathrm{mod}\;\left(n+1\right)$ and $y^{n+1}\in B_{I}\left(x^{n}\right)$. More precisely, we have $g:X^{n}\to {⋃}_{i\geq 0}S_{n+1,(n+1)i}$, where $g\left(x^{n}\right)\in B_{I}\left(x^{n}\right)$. Since $v_{n+1}\left(B_{I}\left(x^{n}\right)\right)=\left\{v_{n}\left(x^{n}\right),\ldots ,v_{n}\left(x^{n}\right)+n+1\right\}$ consists of n+2 consecutive numbers, we can determine $g(x^{n})$ uniquely when $x^{n}\notin VT_{0}\left(n\right)$. On the other hand, if $x^{n}\in VT_{0}\left(n\right)$, both $x^{n}0$ and $x^{n}1$ are possible candidates for $g(x^{n})$. In this case, we set $g\left(x^{n}\right)=x^{n}0$.

Define another function $h:X^{n+1}\to X^{n}$, where $h\left(x^{n+1}\right)=x^{n}$. The function *h* simply deletes the last bit. If we combine two functions, we get $f\left(x^{n}\right)=h\left(g\left(x^{n}\right)\right)$. Then, the function *f* satisfies the following properties.

**Lemma** **6.**

*1*. *For all $x^{n}\in X^{n}$, we have $f\left(x^{n}\right)\in VT_{0}\left(n\right)$.*
*2*. *If $x^{n}\in VT_{0}\left(n\right)$, then $f\left(x^{n}\right)=x^{n}$.*
*3*. *For all $x^{n}\in X^{n}$, we have $|v_{n}\left(f\left(x^{n}\right)\right)-v_{n}\left(x^{n}\right)|\leq n$.*

**Proof.** 

1. Let $y^{n+1}=g\left(x^{n}\right)$, then we have
  $$\begin{array}{c} v_{n}\left(f\left(x^{n}\right)\right)\equiv v_{n}\left(h\left(y^{n+1}\right)\right)\equiv v_{n+1}\left(y^{n+1}\right)\equiv 0\left(\mathrm{mod}\;n+1\right). \end{array}$$
2. If $v_{n}\left(x^{n}\right)\equiv 0\;\mathrm{mod}\;(n+1)$, then we have $g\left(x^{n}\right)=x^{n}0$, and therefore $f\left(x^{n}\right)=x^{n}$.
3. If we let $y^{n+1}=g\left(x^{n}\right)$, then we have
  $$\begin{array}{cc} v_{n}\left(f\left(x^{n}\right)\right)-v_{n}\left(x^{n}\right)= & v_{n}\left(y^{n}\right)-v_{n}\left(x^{n}\right)\\ = & v_{n+1}\left(y^{n+1}\right)-\left(n+1\right)y_{n+1}-v_{n}\left(x^{n}\right)\\ = & v_{n+1}\left(g\left(x^{n}\right)\right)-\left(n+1\right)y_{n+1}-v_{n}\left(x^{n}\right). \end{array}$$
  Recall that we have $v_{n}\left(x^{n}\right)\leq v_{n+1}\left(y^{n+1}\right)\leq v_{n}\left(x^{n}\right)+n+1$. If $v_{n}\left(x^{n}\right)\in VT_{0}\left(n\right)$, then $f\left(x^{n}\right)=x^{n}$, which implies $v_{n}\left(f\left(x^{n}\right)\right)-f_{n}\left(x^{n}\right)=0$. On the other hand, if $v_{n}\left(x^{n}\right)\notin VT_{0}\left(n\right)$, we have $v_{n}\left(x^{n}\right)+1\leq v_{n+1}\left(g\left(x^{n}\right)\right)\leq v_{n}\left(x^{n}\right)+n$. In such case, $v_{n}\left(f\left(x^{n}\right)\right)-v_{n}\left(x^{n}\right)$ achieves the maximum value when $y_{n+1}=0$ and $v_{n+1}\left(g\left(x^{n}\right)\right)=v_{n}\left(x^{n}\right)+n$. In other words,
  $$\begin{array}{c} v_{n+1}\left(g\left(x^{n}\right)\right)-v_{n}\left(x^{n}\right)-\left(n+1\right)y_{n+1}\leq v_{n}\left(x^{n}\right)+n-v_{n}\left(x^{n}\right)=n. \end{array}$$
  On the other hand, it achieves the minimum value when $y_{n+1}=1$ and $v_{n+1}\left(g\left(x^{n}\right)\right)=v_{n}\left(x^{n}\right)+1$. In other words,
  $$\begin{array}{c} v_{n+1}\left(g\left(x^{n}\right)\right)-v_{n}\left(x^{n}\right)-\left(n+1\right)y_{n+1}\geq v_{n}\left(x^{n}\right)+1-v_{n}\left(x^{n}\right)-\left(n+1\right)=-n. \end{array}$$
  Thus, we have
  $$\begin{array}{cc} |v_{n}\left(f\left(x^{n}\right)\right)-v_{n}\left(x^{n}\right)|\leq & n. \end{array}$$
  This concludes the proof.

□

Then, we have a theorem that supports Conjecture 1.

**Theorem** **2.** *For positive integer n, any single deletion code C satisfies the following inequality.*

$$\begin{array}{c} |C_{0}\left|+\right|C_{1}\left|+\cdots \right|C_{n+1}\left|\leq \right|S_{n,0}\left|+\right|S_{n,n+1}|. \end{array}$$

**Proof.**  Let $C^{\prime }={⋃}_{i=0}^{n+1}C_{i}$. From the above lemma, we have $v_{n}\left(f\left(x^{n}\right)\right)\leq v_{n}\left(x^{n}\right)+n\leq 2n+1$ for all $x^{n}\in C^{\prime }$. Since $f\left(x^{n}\right)\in VT_{0}\left(n\right)$, it is clear that $v_{n}\left(f\left(x^{n}\right)\right)$ should be either 0 or n+1. In other words,

$$\begin{array}{c} f\left(x^{n}\right)\in S_{n,0}\cup S_{n,n+1}. \end{array}$$

Suppose there exists $x^{n},{\tilde{x}}^{n}\in C^{\prime }$ such that $f\left(x^{n}\right)=f\left({\tilde{x}}^{n}\right)=y^{n}$, then $v_{n}\left(y^{n}\right)$ is either 0 or n+1. First, if $v_{n}\left(y^{n}\right)=0$, then $y^{n}=0^{n}$. In this case, the weights (number of ones) of $x^{n}$ and ${\tilde{x}}^{n}$ are at most one, i.e., $w\left(x^{n}\right),w\left({\tilde{x}}^{n}\right)\leq 1$. This implies that $0^{n-1}\in B_{D}\left(x^{n}\right)\cap B_{D}\left({\tilde{x}}^{n}\right)$ which is a contradiction. On the other hand, consider the case where $v_{n}\left(y^{n}\right)=n+1$. Since $g\left(x^{n}\right),g\left({\tilde{x}}^{n}\right)\leq 2n+1$ and $v_{n+1}\left(y^{n}1\right)$, we have $g\left(x^{n}\right)=g\left({\tilde{x}}^{n}\right)=y^{n}0$. This implies that $B_{I}\left(x^{n}\right)\cap B_{I}\left({\tilde{x}}^{n}\right)$ has at least one element, and therefore $B_{D}\left(x^{n}\right)\cap B_{D}\left({\tilde{x}}^{n}\right)\neq \emptyset$. This is a contradiction.

Thus, ${f|}_{C^{\prime }}:C^{\prime }\to S_{n,0}\cup S_{n,n+1}$ is an injective function, and therefore

$$\begin{array}{c} |C^{\prime }\left|=\right|C_{0}\left|+\right|C_{1}\left|+\cdots +\right|C_{n+1}\left|\leq \right|S_{n,0}\left|+\right|S_{n,n+1}|. \end{array}$$

□

### 4.4. Integer Programming

As mentioned above, if we can find a single deletion code that satisfies the inequality in Equation (1) or the inequality in Equation (2), that immediately disproves the optimality of VT code. Since the size of the problem is relatively smaller when *k* is small, we can numerically solve the Integer Programming (IP) problem without relaxation.

For example, we can check whether the inequality in Equation (1) holds or not for some fixed *n* and *k*, using the following optimization problem.

$$\begin{array}{cc} \max_{X}\; & \sum_{i:v_{n}\left(b\left(i\right)\right)\leq \left(n+1\right)k}X_{i}\\ s.t.\; & \sum_{i\in Q_{I}\left(y^{n-1}\right)}X_{i}\leq 1,\;\mathrm{for}\;y^{n-1}\in X^{n-1}\\ \; & X_{i}\in \left\{0,1\right\},\;i=0,1,\ldots ,N-1. \end{array}$$

For some *n* and *k*, if the maximum value of the objective function is smaller than or equal to

$$\begin{array}{c} M_{n,k}\overset{\Delta }{=}|S_{n,0}\left|+\right|S_{n,n+1}\left|+\ldots +\right|S_{n,(n+1)k}|, \end{array}$$

then it means that no single deletion code satisfies the inequality in Equation (1).

Note that the number of variables are $\left|\{x^{n}\in X^{n}:v_{n}\left(x^{n}\right)\leq \left(n+1\right)k\}\right|$, which is strictly smaller than $2^{n}$. We solve the above optimization numerically for various *n* and *k*. For all combinations that we tried, the maximum value of the objective function is $M_{n,k}$. The following table shows all combinations of (n,k) that we were able to check in a reasonable amount of time.
n**11****12****13****14****15****16**k=1✓✓✓✓✓✓k=2✓✓✓✓✓✓k=3✓✓✓✓✓✓k=4✓✓✓✓



Entries without check mark are combinations that we could not check due to the running time.

Similarly, we numerically verify the inequality in Equation (2) from Lemma 4. For all combinations of (n,k) that we tried, we were not able to find any counterexample. The following table shows all combinations that we were able to check.
n**11****13****15**k=1✓✓✓k=2✓✓✓k=3✓✓✓

## 5. Conclusions

We investigated the maximum size of binary single deletion code. Tighter upper bounds are provided using Mixed Integer Linear Programming relaxation. In the case of n=11, we showed that the size of the largest single deletion code is smaller than or equal to 173. This implies that the largest single deletion code is size of either 172 or 173. In addition, we showed a conjecture that is equivalent to the optimality of VT code. Numerical results are proposed that support the conjecture that VT code is an optimum single deletion code.

One possible direction for future work is semi-relaxed Semidefinite Programming problem. Since Lovász number (which is based on Semidefinite Programming) provides a better bound than LP, we can propose the Mixed Integer Semidefinite Programming as we semi-relaxed the LP problem. Solvers for Mixed Integer Semidefinite Programming are not as popular as solvers for MILP except a few initial works [31]. However, we think it can verify the optimality of VT code in the case of n=11.
